# Epitaxial Graphene/n-Si Photodiode with Ultralow Dark Current and High Responsivity

**DOI:** 10.3390/nano15151190

**Published:** 2025-08-03

**Authors:** Lanxin Yin, Xiaoyue Wang, Shun Feng

**Affiliations:** 1 College of Information Science and Engineering, Northeastern University, Shenyang 110819, China; 20225363@stu.neu.edu.cn; 2 Shenyang National Laboratory for Materials Science, Institute of Metal Research, Chinese Academy of Sciences, 72 Wenhua Road, Shenyang 110016, China; xywang20s@imr.ac.cn; 3 School of Materials Science and Engineering, University of Science and Technology of China, 72 Wenhua Road, Shenyang 110016, China

**Keywords:** graphene, n-type silicon, heterojunction, photodetector, self-powered operation

## Abstract

Graphene’s exceptional carrier mobility and broadband absorption make it promising for ultrafast photodetection. However, its low optical absorption limits responsivity, while the absence of a bandgap results in high dark current, constraining the signal-to-noise ratio and efficiency. Although silicon (Si) photodetectors normally offer fabrication compatibility, their performance is severely hindered by interface trap states and optical shading. To overcome these limitations, we demonstrate an epitaxial graphene/n-Si heterojunction photodiode. This device utilizes graphene epitaxially grown on germanium integrated with a transferred Si thin film, eliminating polymer residues and interface defects common in transferred graphene. As a result, the fabricated photodetector achieves an ultralow dark current of 1.2 × 10^−9^ A, a high responsivity of 1430 A/W, and self-powered operation at room temperature. This work provides a strategy for high-sensitivity and low-power photodetection and demonstrates the practical integration potential of graphene/Si heterostructures for advanced optoelectronics.

## 1. Introduction

Owing to its exceptional carrier mobility (up to 60,000 cm^2^/(V·s) on a substrate and at room temperature [1,2]) and broadband spectral response spanning ultraviolet (UV) to terahertz [2,3,4,5], graphene is widely regarded as a promising material for ultrafast and broadband photodetection [6,7,8,9,10,11,12,13,14,15,16]. To date, various graphene-based photodetector architectures (e.g., metal-graphene junctions [6,8,16], and graphene p-n junctions [17,18,19,20]) have been demonstrated. Relying on the unique electronic properties of graphene, these devices can achieve broadband detection with ultrafast response. However, two intrinsic limitations hinder the application of graphene-based photodetectors. First, the intrinsically low optical absorption of graphene (monolayer, ~2.3% [21]) severely limits the generation of photogenerated carriers, resulting in the responsivity typically on the order of mA/W [15], significantly lower than commercial standards [22]. On the other hand, the zero bandgap nature of graphene prevents effective suppression of the hot carriers, leading to high dark current [18,23]. Consequently, the elevated dark current degrades the signal-to-noise ratio (SNR) and reduces the energy conversion efficiency [24].

In contrast, silicon (Si)-based photodetectors have dominated the field of optoelectronics for decades due to their seamless integration with the complementary metal-oxide-semiconductor (CMOS)-compatible fabrication process [15,25]. However, as an indirect bandgap semiconductor, Si exhibits a relatively low intrinsic optical absorption coefficient, which significantly limits the photoelectric conversion efficiency of Si photodetectors [22]. To address this limitation, various Si micro/nanostructures, such as Si nanowire, micro/nanoporous Si, and micropyramid Si, have been developed to enhance light absorption [26,27,28,29]. These structures improve the optical path length and reduce the surface reflection, thereby improving the photoelectric energy conversion efficiency. Nevertheless, the conventional fabrication process of Si-based photodetectors still relies on the deposition of metal electrodes, which inevitably induces the optical shading and reduces the effective illumination area [22]. Moreover, the metal–silicon interface is susceptible to the formation of trap states, leading to pronounced carrier recombination and reduced responsivity [22].

To overcome these limitations, graphene has emerged as a promising transparent electrode material in Si-based photodetectors, enabling the formation of graphene/Si Schottky junctions [30,31,32]. Unlike conventional metal electrodes, graphene offers exceptional optical transmittance [21], high carrier mobility [1,2], and a tunable Fermi level [33]. These properties make it highly attractive for a wide range of applications, including photodetection, solar harvesting, and sensing. In particular, graphene/Si photodiodes demonstrate commercial-level responsivity across UV-visible spectra, while also offering advantages in scalability and simplified fabrication [34,35]. However, prevailing fabrication approaches for such photodiodes still suffer from critical issues, including polymer residues introduced during the wet transfer of graphene and surface/interface defects caused by micro/nanostructure etching [26]. These imperfections contribute to increased dark current, thereby significantly degrading device performance in terms of SNR, responsivity, and power efficiency [36].

To address these challenges, we reported a graphene/n-type silicon (n-Si) heterojunction photodiode via in situ growth of graphene on germanium (Ge) substrates [37], followed by the transfer of Si thin film onto the graphene/Ge stack. This fabrication strategy effectively eliminates interface defects and polymer residues typically introduced during conventional etching and transfer processes. As a result, the graphene/n-Si photodiode achieves an ultralow dark current of 1.2 × 10^−9^ A and a high responsivity of 1430 A/W. In addition, the device delivers a large short-circuit current of 5.6 × 10^−6^ A, enabling an efficient self-powered operation without external bias.

## 2. Methods

### 2.1. Graphene Grown Epitaxially on Ge

A CVD method was applied to grow the high-quality graphene on a 4-inch n-type <110> Ge substrate. The Ge substrate was placed in a 4-inch horizontal quartz tube with the vacuum degree about 0.1 Pa. After the quartz tube was heated to 916 °C and filled with high purity argon (700 sccm) and hydrogen (70 sccm), CH_4_ (2.2 sccm) was introduced to grow graphene on the Ge substrate. Finally, a 50 nm Ti/Au electrode was evaporated on top of the graphene by photolithography and electron beam evaporation.

### 2.2. Transfer of the n-Si Film

The n-Si film was achieved from a silicon-on-insulator (SOI) substrate with a 3 μm-thick top single-crystal Si layer (resistivity: 1–20 Ω cm), and a 0.5 μm-thick buried SiO_2_ layer. First, a 50 nm-thick Ti/Au electrode was deposited on the surface of top Si layer of SOI by electron beam evaporation. Then the Si layer with top electrode was patterned into 30 × 30 μm^2^ square-shaped film by RIE with the CF_4_ and O_2_. Next, 40 wt% concentrated HF was used to etch away the buried SiO_2_, leaving the patterned top Si film onto the SOI substrate. Finally, PDMS film was placed on the SOI substrate to retrieve the Si film and peeled away quickly. To release the Si film, the PDMS was then placed on the target substrate and peeled away slowly, leaving the Si membrane onto the graphene.

## 3. Characterization

The devices were characterized using an optical microscope (LV100ND, Nikon, Tokyo, Japan), an AFM (Bruker Dimension Icon, Bruker, Billerica, MA, USA), an SEM (ZEISS Sigma 300, Carl Zeiss AG, Oberkochen, Germany), and a micro-Raman analyzer (Jobin Yvon HR800, Horiba, Kyoto, Japan). The electrical and optoelectronic performances were measured using a semiconductor analyzer (Agilent B1500A, Agilent Technologies, Santa Clara, CA, USA), a probe station (Cascade M150, FormFactor, Livermore, CA, USA), and a laser diode controller (ITC4001, Thorlabs, Newton, MA, USA), using laser excitations of 405, 516, 638, and 800 nm in a dark room at room temperature.

## 4. Results

**Figure 1:**
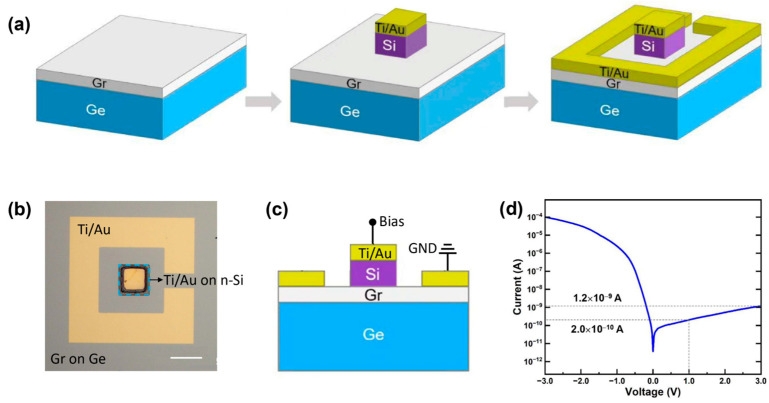
Device structure and fabrication. (**a**) Illustration of the fabrication process of the graphene (Gr)/n-Si photodiode. (**b**) Optical image (scale bar: 50 μm) of the graphene/n-Si photodiode. (**c**) Cross-sectional schematic diagram of the device. (**d**) Current–voltage (*I*–*V*) curve of the device in the dark. The dark current values at 1 V and 3 V are given.

As illustrated in Figure 1a, the graphene/n-Si heterojunction photodetector consists of four primary components: gold (Au) electrodes, n-Si, graphene, and Ge substrate. The detailed fabrication process involved four sequential steps. The first step is the pretreatment of Ge substrate and the growth of graphene [37]. Specifically, the Ge substrate is sequentially cleaned by ultrasonic cleaning in acetone, isopropanol, and deionized water to remove surface contaminants. This is followed by oxide removal using a diluted hydrofluoric acid (HF) solution. Subsequently, high-quality single-crystal monolayer graphene is grown epitaxially on the Ge substrate via a chemical vapor deposition (CVD) process. The second step is patterning the n-Si film. This process begins with the photolithographic definition of electrode patterns on a SiO_2_/Si substrate, followed by the deposition of Ti/Au electrodes via electron beam evaporation. Subsequently, reactive ion etching (RIE) is employed to isolate the n-Si film region. The third step is the transfer of the n-Si film. This is accomplished by etching the SiO_2_ sacrificial layer using HF, followed by a solid polydimethylsiloxane (PDMS) assisted lift-off to transfer the n-Si film onto the graphene/Ge substrate. The final step is the fabrication of graphene electrode. In this step, photolithography is again employed to define a photoresist mask layer, exposing the desired electrode regions on the graphene layer. Ti/Au electrodes are then deposited via electron beam evaporation to complete the device structure.

Figure 1b shows the optical micrograph of the fabricated device, highlighting the 30 × 30 μm^2^ n-Si membrane. The cross-sectional schematic of this graphene/n-Si heterojunction structure is shown in Figure 1c, clearly illustrating the device architecture. The morphological and structural characterizations of devices are also shown in Figure S1. The scanning electron microscopy (SEM) image (Figure S1a) shows that the device surface is extremely clean, without visible contamination or residues, ensuring a pristine graphene/n-Si heterojunction interface. The atomic force microscopy (AFM) measurements (Figure S1b) indicate that the root-mean-square roughness (Ra) of the epitaxial graphene on Ge substrate is only 1.26 nm, confirming its high surface flatness and uniformity. Such smooth graphene morphology is beneficial for minimizing interface trap states and enhancing charge transport. While the Raman scattering spectra (Figure S1c) of epitaxial monolayer graphene on n-Ge exhibit two characteristic peaks, the G band at 1580 cm^−1^ and the 2D band at 2700 cm^−1^, the D band associated with defects is absent. All these results clearly demonstrate a high crystallinity and a low defect density of the epitaxial graphene on Ge.

To evaluate the rectifying behavior of the graphene/n-Si photodiode, the dark current–voltage (*I*–*V*) curve was measured, which demonstrates an excellent diode-like rectification, achieving a rectification ratio up to 105 (Figure 1d). Notably, the dark current remains as low as 2.0 × 10^10^ A at a bias of 1 V and only increases to 1.2 × 10^−9^ A at 3 V, highlighting the ultralow dark current of the device.

**Figure 2:**
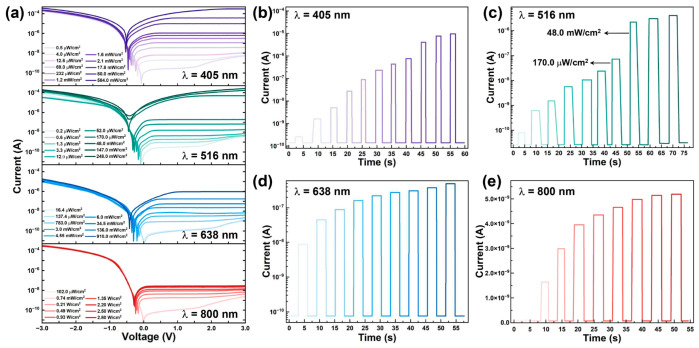
Photoresponse characterization of the device. (**a**) –*V* curves of the device under illumination at different wavelengths (405 nm, 516 nm, 638 nm, and 800 nm) and various light intensities. Time-resolved photoresponse of the device under zero bias upon illumination with (**b**) 405 nm, (**c**) 516 nm, (**d**) 638 nm, and (**e**) 800 nm lasers at varied light intensities. The detailed light intensity values for the step-like change in current are explicitly given in (**c**) for better understanding.

To systematically evaluate the photoelectric characteristics of the fabricated graphene/n-Si heterojunction photodetector across a broad spectral range, the device was illuminated with laser sources of varying wavelengths spanning from UV to near-infrared. Specifically, laser beams at 405 nm, 516 nm, 638 nm, and 800 nm wavelengths were employed to conduct *I*–*V* measurements under different illumination intensities. As a result, according to the corresponding *I*–*V* curves shown in Figure 2a, the graphene/n-Si photodiode exhibits significant photoresponse across all tested wavelengths, while the reverse current increases markedly with the incident light intensity, suggesting an efficient photogenerated carrier separation at the graphene/n-Si junction. In addition, as increasing the light intensity, the open-circuit voltage (VOC, defined as the bias at which the photogenerated current offsets the forward dark current in the *I*–*V* curve) gradually shifts towards the forward bias direction, further confirming the enhancement of the built-in photovoltage.

**Figure 3:**
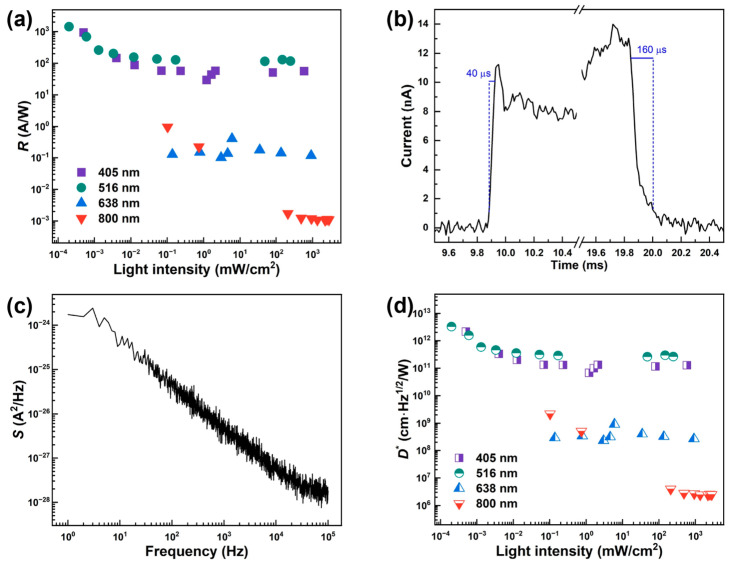
Performance evaluation of the device. (**a**) Photoresponsivity (*R*) as a function of incident light intensity under four laser wavelengths (405, 516, 638, and 800 nm). (**b**) Time-resolved photoresponse of the Gr/n-Si photodetector. Rise and fall times are defined as the time required to switch between 10% and 90% of the maximum photocurrent. (**c**) Density spectral density (S) as a function of frequency. (**d**) Specific detectivity (D*) as a function of incident light intensity under four laser wavelengths (405, 516, 638, and 800 nm).

Furthermore, to evaluate the self-powered capability of the graphene/n-Si heterojunction photodiode, we conducted time-resolved photoresponse measurements under open-circuit conditions using pulsed laser sources with various wavelengths (405 nm, 516 nm, 638 nm, and 800 nm) and light intensities. As shown in Figure 2b–e, a 3 s light pulse was applied to trigger the device response, and the resulting photocurrent was recorded in real time. Consistent with the *I*–*V* measurements, the device exhibits a clear intensity-dependent photoresponse across the full spectral range, reaffirming its excellent photocarrier generation and separation efficiency. Notably, under 516 nm laser illumination at an intensity of 450 mW/cm^2^, the graphene/n-Si photodiode achieves a peak self-powered photocurrent of 5.6 × 10^−6^ A (Figure 2c), corresponding to a responsivity up to 116 A/W (Figure 3a). In addition, the device demonstrates a fast photoresponse with a rise time of 40 μs and a fall time of 160 μs (Figure 3b), indicating a rapid carrier dynamics and the minimal trapping effects under zero bias conditions. These results demonstrated a robust response of the device under zero external bias, underscoring its potential for high-sensitivity, energy-efficient, and self-driven optoelectronic applications.

To quantitatively assess the photoresponse performance, we calculated the photoresponsivity (*R*) of the graphene/n-Si heterojunction photodiode under illumination with various laser wavelengths at a bias of 3 V. The photoresponsivity is defined as [38]

$$R=\frac{I_{light}-I_{dark}}{P_{in}\;\times \;S}$$

where $I_{dark}$ and $I_{light}$ represent the current in dark (Figure 1d) and under light illumination (Figure 2a), respectively, while $P_{in}$ is the power intensity of incident light, and S is the effective illuminating area (30 × 30 μm^2^ for this device). As shown in Figure 3a, the graphene/n-Si photodiode exhibits high responsivity, reaching 939 A/W at 405 nm and 1430 A/W at 516 nm. This enhanced performance at shorter wavelengths can be attributed to the higher photon energy, which facilitates the generation of a greater density of electron–hole pairs within the Si absorber [39]. Owing to this advantage, the device shows great promise in application scenarios requiring high short-wavelength sensitivity, such as green-light hemoglobin detection and UV monitoring [40]. The wavelength-dependent responsivity of the fabricated graphene/n-Si photodiode is shown in Figure S2a. As the incident light shifts from the visible region to the near-infrared region, the device exhibits a pronounced decrease in responsivity. This reduction can be mainly attributed to the significantly lower optical absorption coefficient of silicon in the near-infrared range, which leads to fewer photogenerated carriers and thus weaker photocurrent. In order to demonstrate the device testing statistics, we have tested six more devices. As shown in Figure S2b, all these six devices (#1–6) exhibit a very consistent result for the responsivity (ranging from 47.5 to 95.2 A/W at 405 nm with a light intensity of 584 mW/cm^2^, well consistent with the value (56.2 A/W at 405 nm with a light intensity of 584 mW/cm^2^) presented in Figure 3a. Additionally, these six devices (#1–6) also exhibit a very similar behavior of the time-resolved photoresponse under zero bias upon illumination with a 405 nm laser at varying light intensities, as shown in Figure S3. These well-calibrated data further validate the superiority and excellent performance of the graphene/n-Si heterojunction photodiode fabricated in this work, demonstrating its fast response speed and high operational stability.

The noise spectral density as a function of frequency at an applied bias of 3 V is presented in Figure 3c. The low-noise spectrum exhibits a typical 1/f power dependence, which is the characteristic of high-quality photodiodes with minimal flicker noise. Based on the measured noise data and the responsivity, we further calculated the specific detectivity (D*) of the graphene/n-Si photodiode using the standard equation: D* = (*AB*)1/2*R*/*S*1/2, where *A* is the active area of 900 μm^2^, *B* is the bandwidth (1 Hz), *R* is the responsivity, and *S* is the noise spectral density of the device. The detectivity exhibits the same wavelength-dependent trend as the responsivity, reaching a maximum value of 3.26 × 1012 cm·Hz1/2/W under illumination with 516 nm laser, demonstrating the excellent photodetection performance of this graphene/n-Si device, which is comparable to or even higher than previously reported graphene/silicon photodiodes (Table 1).

**Figure 4:**
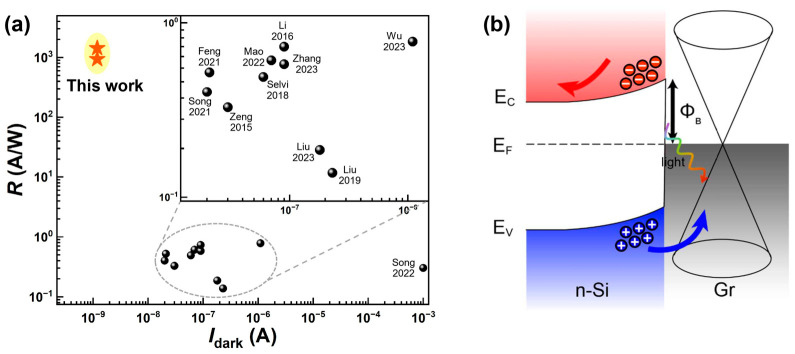
Benchmarking and mechanism analysis of the device. (**a**) Benchmark comparison of Si-based photodetectors plotted as photoresponsivity (*R*) versus dark current ($I_{dark}$). The data of other reported Si-based photodiodes are cited from Mao 2022 [26], Wu 2023 [29], Li 2016 [32], Selvi 2018 [36], Zhang 2023 [38], Zeng 2015 [42], Liu 2019 [43], Feng 2021 [44], Song 2022 [45], Liu 2023 [46], and Song 2021 [47]. (**b**) Energy band diagrams of the graphene/n-Si heterojunction. E_C_ and E_V_ denote the conduction band and valence band of n-Si, E_F_ denotes the Fermi level, Φ_B_ denotes the Schottky barrier of the device. The red and blue arrows denote the transport paths of photogenerated electrons and holes, respectively. The black up-down arrow denotes the height of the Schottky barrier. The colorful crooked arrow illustrates the light illumination.

To further validate the performance of our device, we benchmarked it against other reported Si-based Schottky photodiodes (Figure 4a). As shown here, our device exhibits an ultralow dark current of 1.2 × 10^−9^ A at a forward bias of 3 V, substantially lower than comparable counterparts, underscoring its excellent power efficiency. Meanwhile, the responsivities of 939 A/W at 405 nm and 1430 A/W at 516 nm significantly exceed those of most existing devices, highlighting the superior photodetection capabilities of the proposed graphene/n-Si heterojunction photodiode. Compared with other reported Si-based photodiodes, the much higher performance of our graphene/n-Si device can be mainly ascribed to the direct utilization of epitaxial graphene grown on Ge substrate. As demonstrated by the morphological and structural characterizations (Figure S1), such an epitaxial graphene on Ge features high crystallinity and high surface flatness. In this case, high interface quality can be achieved between graphene and n-Si, thus leading to an extremely high photodetection performance of our graphene/n-Si device. In fact, based on epitaxial graphene on Ge, an atomically clean and sharp interface was observed between graphene and gold for the gold/graphene/germanium photodetector reported previously by our group [41], which more clearly demonstrates the significant advantages of our graphene/n-Si photodiode over previously reported Si-based devices.

**Table 1:** Performance of Si-based photodiodes.

Device Structure	Responsivity (A/W)	Specific Detectivity (cm·Hz1/2/W)	*I_dark_*	Reference
graphene	0.78		1.1 µA at −1.0 V	[29]
MoS_2_/Si	0.61		70 nA at −1.0 V	[26]
Si/In_2_Se_3_	0.58		90 nA at −3.0 V	[38]
Graphene/n-Si	0.73		90 nA at −0.4 V	[32]
Ti_3_C_2_/Si	0.402	2.05 × 10^13^	20 nA at −1.0 V	[47]
Bilayer-graphene/Si	0.328		30 nA at −0.5 V	[42]
Graphene/Au/Si	0.138	1.40 × 10^10^	230 nA at −1.0 V	[43]
Graphene/Si	0.52	1.43 × 10^13^	21 nA at −0.4 V	[44]
Ti_3_C_2_T_x_/Si	0.302	5.40 × 10^13^	1.0 mA at −2.0 V	[45]
V_2_CT_x_/n-Si	0.187	8.42 × 10^11^	180 nA at −3.0 V	[46]
Graphene/Si	0.49		60 nA at +1.5 V	[36]
Graphene/n-Si	1430	3.26 × 10^12^	1.2 nA at +3.0 V	This work

The device structure, responsivity, and dark current of relevant counterparts are also summarized in Table 1.

Furthermore, to gain deeper insight into the photodetection behavior of the graphene/n-Si heterojunction, we analyzed its working mechanism based on band structure considerations. As shown in Figure 4b, the formation of the graphene/n-Si heterojunction induces an upward band bending in n-Si near the interface, leading to the creation of a depletion region with a built-in electric field. This built-in field facilitates the separation of photogenerated electron–hole pairs, even in the absence of external bias, forming the basis of the device’s self-powered photoresponse. Under forward bias, the depletion region narrows and the energy bands shift upward, effectively lowering the Schottky barrier height. This change enhances carrier injections across the junction, thereby increasing the current and enabling diode-like conduction. In contrast, applying a reverse bias widens the depletion region and shifts the bands downward, which raises the effective barrier height and restricts carrier transport. These behaviors are consistent with the *I*–*V* curves presented in Figure 1d and Figure 2a.

Critically, epitaxial graphene growth on Ge substrate avoids transfer-induced defects, thereby forming a high-quality Schottky interface with reduced defect density and suppressed dark current. Upon illumination, photon absorption in n-Si generates electron–hole pairs. The built-in electric field within the depletion region efficiently separates carriers: photoexcited holes are swiftly injected into the graphene layer, while electrons are driven into the silicon bulk. In addition, holes generated in the quasi-neutral region of n-Si diffuse toward the depletion region and are subsequently swept into graphene by the electric field, further enhancing carrier separation efficiency. This dynamic leads to the accumulation of holes in graphene and electrons in silicon, respectively, thus establishing a photovoltage across the junction. When an external circuit is connected, electrons flow from the silicon side to recombine with holes in graphene, resulting in a net photocurrent directed from graphene to n-Si.

## 5. Conclusions

In conclusion, we reported a high-performance graphene/n-Si photodiode fabricated by transferring a Si film onto graphene epitaxially grown on Ge substrate. This approach enables the formation of an atomically clean heterojunction interface, effectively suppressing defects and impurity states, thereby minimizing reverse leakage current. As a result, this graphene/n-Si heterojunction photodetector exhibits three key advantages: (1) an ultralow dark current of 1.2 × 10^−9^ A at 3 V bias for exceptional power efficiency; (2) a high responsivity of 1430 A/W at 516 nm, surpassing most Si-based photodetectors; and (3) a substantial self-powered current of 5.6 × 10^−6^ A at 0 V bias, confirming reliable bias-free operation. Benefiting from the combination of low dark current, high responsivity, and self-powering capability, this graphene/n-Si heterojunction photodiode holds great promise for broadband, high-sensitivity photodetection, particularly in self-driven systems without power supply. Moreover, the fabrication process is highly compatible with standard Si-based chip manufacturing, offering a practical and scalable route toward next-generation integrated optoelectronic systems with low power consumption and high performance.

## Data Availability

The data presented in this study are available upon request.

## Supplementary Materials

The following supporting information can be downloaded at https://www.mdpi.com/article/10.3390/nano15151190/s1, Figure S1. The morphological and structural characterizations of the fabricated graphene/n-Si device, including the SEM image of the device and the AFM image and the Raman scattering spectra of epitaxial graphene on Ge substrate. Figure S2. The wavelength-dependent responsivity of the fabricated Gr/n-Si device and the responsivities of six fabricated Gr/n-Si devices (device #1–6) under 405 nm laser illumination at a light intensity of 584 mW/cm^2^. Figure S3. Time-resolved photoresponse of six devices (device #1–6) under zero bias upon illumination with 405 nm laser at ten varied light intensities.
