# Supplementary material for: Epitaxial Graphene/n-Si Photodiode with Ultralow Dark Current and High Responsivity
Source: Nanomaterials (Basel). 2025 Aug 3;15(15):1190. doi: 10.3390/nano15151190 (PMC12348325; doi:10.3390/nano15151190)
Supplement: Supplementary file 1 [file nanomaterials-15-01190-s001.zip › nanomaterials-3761271-supplementary.pdf]

Supporting Information

Epitaxial graphene/n-Si photodiode with ultralow dark current and high responsivity

Lanxin Yin [1,†], Xiaoyue Wang [2,3,†], Shun Feng [2,3,*]

[1]College of Information Science and Engineering, Northeastern University, Shenyang, 110819, China; 20225363@stu.neu.edu.cn (L.Y.)

[2]Shenyang National Laboratory for Materials Science, Institute of Metal Research, Chinese Academy of Sciences, 72 Wenhua Road, Shenyang 110016, China; xywang20s@imr.ac.cn (X.W.); sfeng@imr.ac.cn (S.F.)

[3]School of Materials Science and Engineering, University of Science and Technology of China, 72 Wenhua Road, Shenyang 110016, China

[*] Correspondence: sfeng@imr.ac.cn

[†] These authors contributed equally to this work.

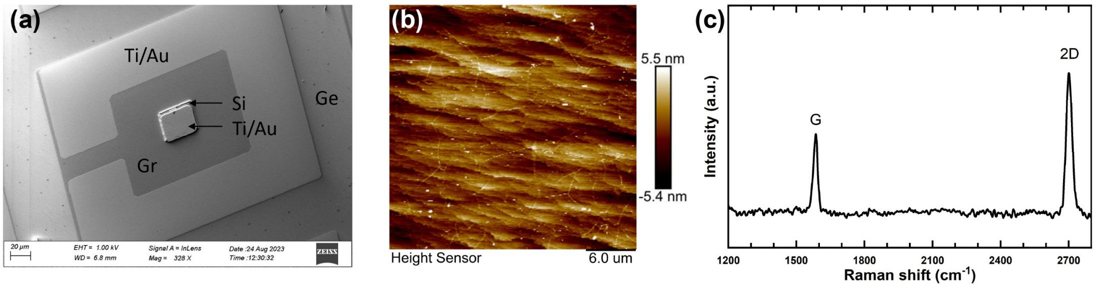

Figure S1. The morphological and structural characterizations of the fabricated graphene/n-Si device. (a) SEM image of the fabricated Gr/n-Si device (scale bar: 20 μm). (b) AFM image of epitaxial graphene on n-Ge in the height sensor mode (scale bar: 6 μm), showing a Ra value of 1.26 nm. (c) Raman scattering spectra of epitaxial graphene on n-Ge.

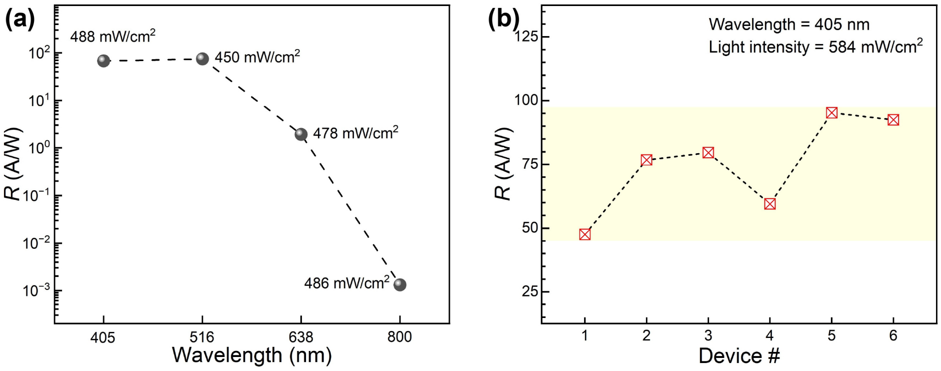

Figure S2. (a) The wavelength-dependent responsivity of the fabricated Gr/n-Si device. Note that, it is very difficult to adjust the light intensity of lasers with different wavelengths to be an exactly the same value in experiment. (b) The responsivities of six fabricated Gr/n-Si devices under 405 nm laser illumination at a light intensity of 584 mW/cm^2.

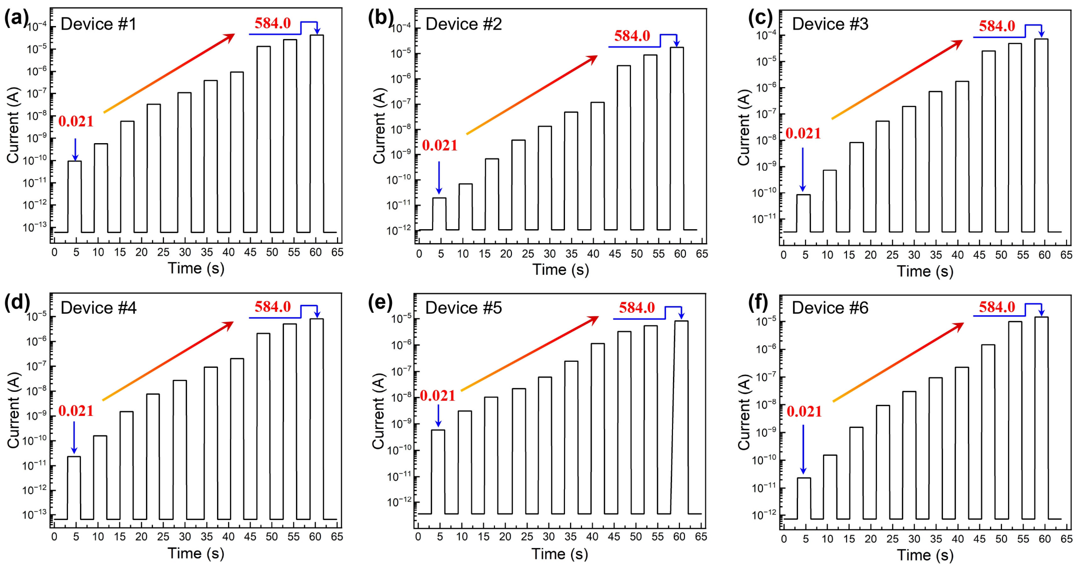

Figure S3. Time-resolved photoresponse of six devices (device #1-6) under zero bias upon illumination with 405 nm laser at ten varied light intensities (0.021, 0.066, 0.45, 2.0, 7.8, 28.5, 67.0, 259.0, 387.0 and 584.0 mW/cm^2).
